# Determining spatio-temporal characteristics of coseismic travelling ionospheric disturbances (CTID) in near real-time

**DOI:** 10.1038/s41598-021-99906-5

**Published:** 2021-10-21

**Authors:** Boris Maletckii, Elvira Astafyeva

**Affiliations:** CNRS UMR 7154, Institut de Physique du Globe de Paris (IPGP), Université de Paris, 35-39 Rue Hélène Brion, 75013 Paris, France

**Keywords:** Natural hazards, Space physics, Geophysics

## Abstract

Earthquakes are known to generate ionospheric disturbances that are commonly referred to as co-seismic travelling ionospheric disturbances (CTID). In this work, for the first time, we present a novel method that enables to automatically detect CTID in ionospheric GNSS-data, and to determine their spatio-temporal characteristics (velocity and azimuth of propagation) in near-real time (NRT), i.e., less than 15 min after an earthquake. The obtained instantaneous velocities allow us to understand the evolution of CTID and to estimate the location of the CTID source in NRT. Furthermore, also for the first time, we developed a concept of real-time travel-time diagrams that aid to verify the correlation with the source and to estimate additionally the propagation speed of the observed CTID. We apply our methods to the Mw7.4 Sanriku earthquake of 09/03/2011 and the Mw9.0 Tohoku earthquake of 11/03/2011, and we make a NRT analysis of the dynamics of CTID driven by these seismic events. We show that the best results are achieved with high-rate 1 Hz data. While the first tests are made on CTID, our method is also applicable for detection and determining of spatio-temporal characteristics of other travelling ionospheric disturbances that often occur in the ionosphere driven by many geophysical phenomena.

## Introduction

It is known that natural hazard events, such as earthquakes, tsunamis and/or volcanic eruptions generate acoustic and gravity waves that propagate upward in the atmosphere and ionosphere (e.g.,^1–7^). Earthquake-driven ionospheric disturbances are called co-seismic travelling ionospheric disturbances (CTID). The first CTID are generated directly by the ground or the seafloor via acoustic waves, they reach the ionospheric altitudes (~ 200–350 km) in only 7–9 min. They are followed by acoustic waves generated by the surface Rayleigh waves, and tsunami gravity waves. Nowadays, with the development of permanent networks of dual-frequency Global Navigation Satellite Systems (GNSS) receivers, the detection of CTID and other Natural-Hazard-driven (NH-driven) ionospheric perturbations has nowadays become quite regular (e.g.,^5,8–12^).

Recently, it has been suggested that NH-driven ionospheric disturbances can be used for more advanced purposes: to localize NH and to estimate the characteristics of the source (e.g.,^13–19^). Kamogawa et al*.*^20^ suggested a method based on observations of a “tsunami-ionospheric hole”, ionospheric depletion that often occurs after major earthquakes over the epicentral area. Based on the analysis of seven tsunamigenic earthquakes in Japan and Chile, Kamogawa et al*.*^20^ found a quantitative relationship between the initial tsunami height and the TEC depression rate. Manta et al*.*^21^ developed a new ionospheric tsunami power index based on measurements of CTID. They showed that the ionospheric index scales with the volume of water displaced due to an earthquake. However, neither of these methods is real-time compatible. As near-real-time (NRT) mode, we refer to as 10–15 min after an earthquake. Going further towards NRT, Savastano et al*.*^22^ made the first preliminary feasibility demonstration for ionospheric monitoring by GNSS, by developing a software VARION that can derive TEC in NRT. Their technique has been implemented at several GNSS-receivers around the Pacific Ocean (https://iono2la.gdgps.net), and is aiming—in the future—to detect traveling ionospheric disturbances (TID) associated with tsunamis. Shrivastava et al*.*^23^ demonstrated the possibility of tsunami detection by GPS-derived TEC, however, no discussion on the real-time use was provided.

Ravanelli et al*.*^24^ claimed to provide the first real-time ionosphere-based tsunami risk assessment by data GNSS receivers in Chile. However, they analyse 2 h of data and used 8th order polynom, i.e., their approach requires stacking of about 2 h of data. Therefore, this approach is not NRT-compatible by our definition.

Therefore, recent seismo-ionospheric results show a big potential for the future use of ionospheric measurements for natural hazard risk assessment. However, before such methods could be applied in real-time, several major developments are yet to be implemented. Going toward real-time applications, the first step is to automatically detect CTID in near-real-time and to analyze their features in order to prove their relation to earthquakes. In this work, we introduce, for the first time, near-real-time compatible methods for determining the spatio-temporal characteristics of CTID.

## Methods

### Estimation of total electron content (TEC) from GNSS

**Figure 1:**
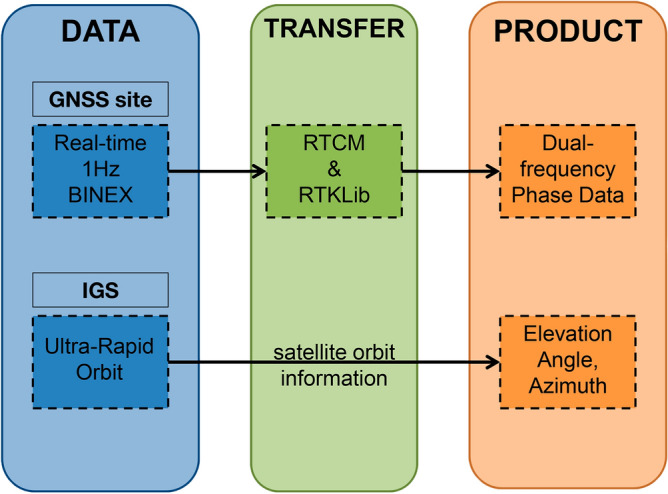
Real-time collection of GNSS phase data and orbit parameters. Networked Transport of RTCM^26^ via Internet Protocol (NTRIP)^27^ could be used to provide the real-time data stream from  GNSS stations. The main goal of the protocol is Real Time Kinematics (RTK), but it is also suitable for our purposes since it transfers dual-frequency phase and pseudo-range data in real time. RTKLib^28^ software could be used to convert binary information from NTRIP data stream. The International GNSS Service (IGS) ultra-rapid orbit^29^ is used to obtain the information about the elevation angle and the azimuth. *BINEX* Binary INdependent EXchange format for files that is used in real-time.

GNSS allows to estimate the ionospheric total electron content (TEC), which is an integral parameter equal to the number of electrons along a line-of-sight (LOS) between a satellite and a receiver. The LOS TEC is often called slant TEC (sTEC). The TEC is usually measured in TEC units (TECU), with 1 TECU equal to 10^16^ electrons/m^2^. To calculate the TEC, one needs phase and code measurements performed by dual-frequency receivers (i.e.,^25^). However, the code measurements are only needed to remove the inter-frequency bias. While, the co-seismic signatures and other disturbances can be retrieved from phase TEC estimated solely from phase measurements:

1 $$sTEC\_ph = \frac{1}{A} \cdot \frac{{f_{1}^{2} f_{2}^{2} }}{{f_{1}^{2} - f_{2}^{2} }}\left( {L_{1} \lambda_{1} - L_{2} \lambda_{2} } \right)$$

where *A* = 40.308 m^3^/s^2^, *L*_1_ and *L*_2_ are phase measurements, *λ*_1_ and *λ*_2_ are wavelengths at the two Global Positioning System (GPS) frequencies (1575,42 and  1227,60 MHz). Therefore, in near-real-time approach, we will only use these phase measurements that can be easily transferred in very short time (Fig. 1). The first data point is removed from the whole data series as the unknown bias.

In order to determine the position of ionospheric disturbances, we estimate the coordinates of so-called sub-ionospheric points (SIP) that represent the intersection points between the LOS and the ionospheric thin shell. The satellite orbit information can be rapidly transferred in NRT from the IGS in navigation RINEX files (Fig. 1), or it can be forecasted very precisely based on the current known satellite coordinates. Otherwise, ultra-rapid orbits can be used. The shell altitude *Hion* is not known but presumed from physical principles: we expect the observed perturbation to be concentrated at the altitude of the ionization maximum (HmF2). In NRT, the value of HmF2 can be obtained either from nearest ionosonde stations, or from empirical ionospheric models, such as NeQuick^30^ or International Reference Ionosphere (IRI)^31^. Here we take *Hion* = 250 km, which is close to the HmF2 on the days of the earthquakes^15,32^.

It should be noted that in the vast majority of previous studies of ionospheric response to earthquakes the researchers used band-pass filters, such as running mean, polynomial fitting, high order Butterworth, etc. (e.g.,^33–35^). However, in a real-time scenario one cannot use such filters because of the impossibility to stack long series of data (up to 30–60 min) and due to the lack of time. In addition, the band-pass filtering would induce artefacts and will affect the properties of the detected signals (arrival time, amplitude, spectral components). Therefore, here we suggest to analyze the rate of TEC change (dTEC/dt) instead of the sTEC. Such a derivative procedure works as a high-pass filter and removes the bias and trend caused by the satellite orbit motion. In addition, our dTEC/dt approach will not modify the amplitude of CTID.

Below we use 1 Hz GNSS data for our real-time scenario.

### Real-time detection of co-seismic travelling ionospheric disturbances from TEC data series

**Figure 2:**
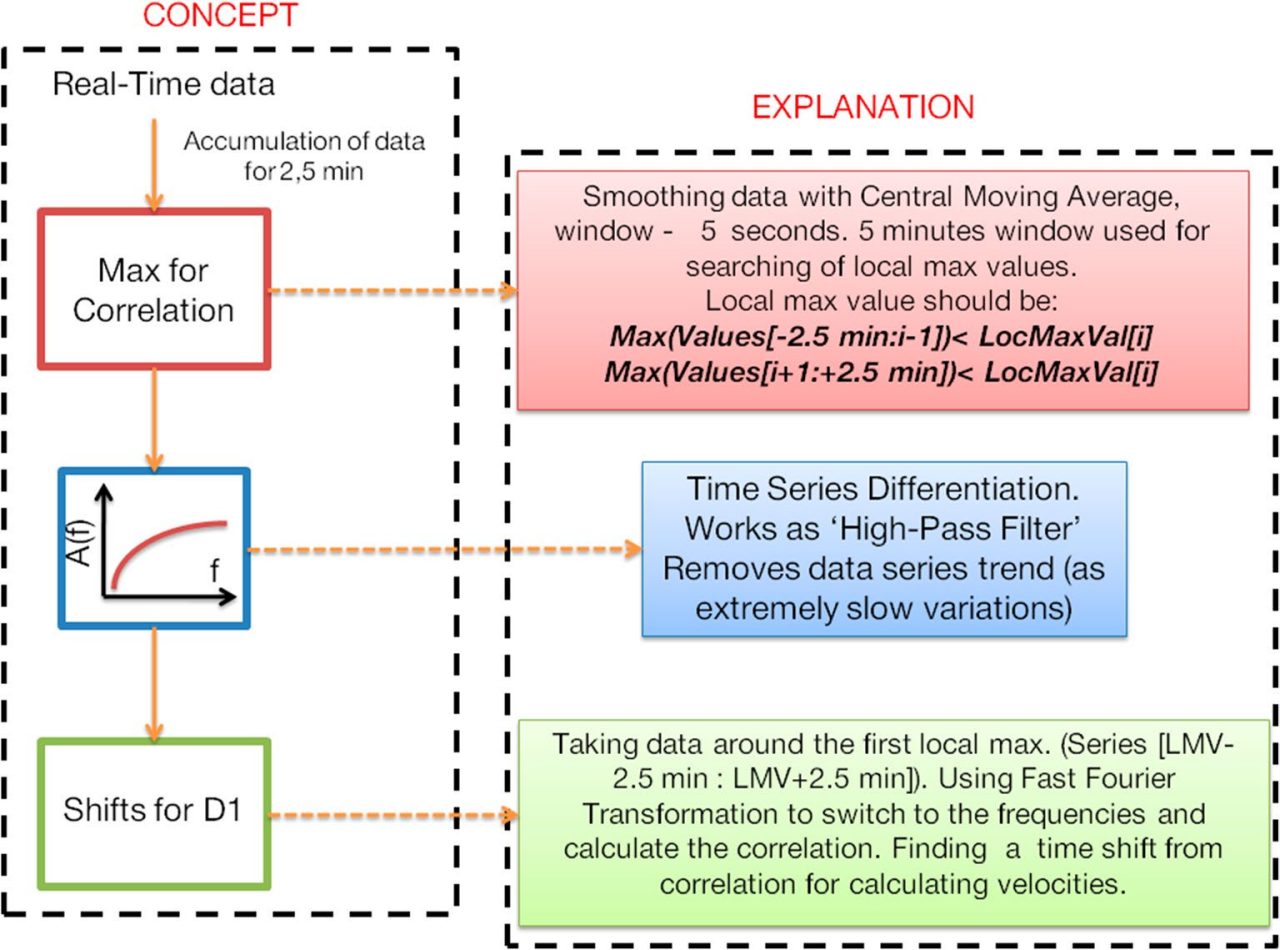
The concept of the near-real-time detection of CTID and TID, and explanation of the main steps of the procedure.

The concept of the developed method is presented in Fig. 2. CTID are detected by analysing the sTEC data series by 5-s centered moving averaged over a 5-min window. The averaging prevents detection of random peaks in data. The window duration is chosen to be NRT-compatible and, at the same time, it allows more thorough analysis of CTID characteristics in multiple data series at later steps. Within the selected time window, we search for a local maximum value (LMV) that must exceed every other value within the window.

**Figure 3:**
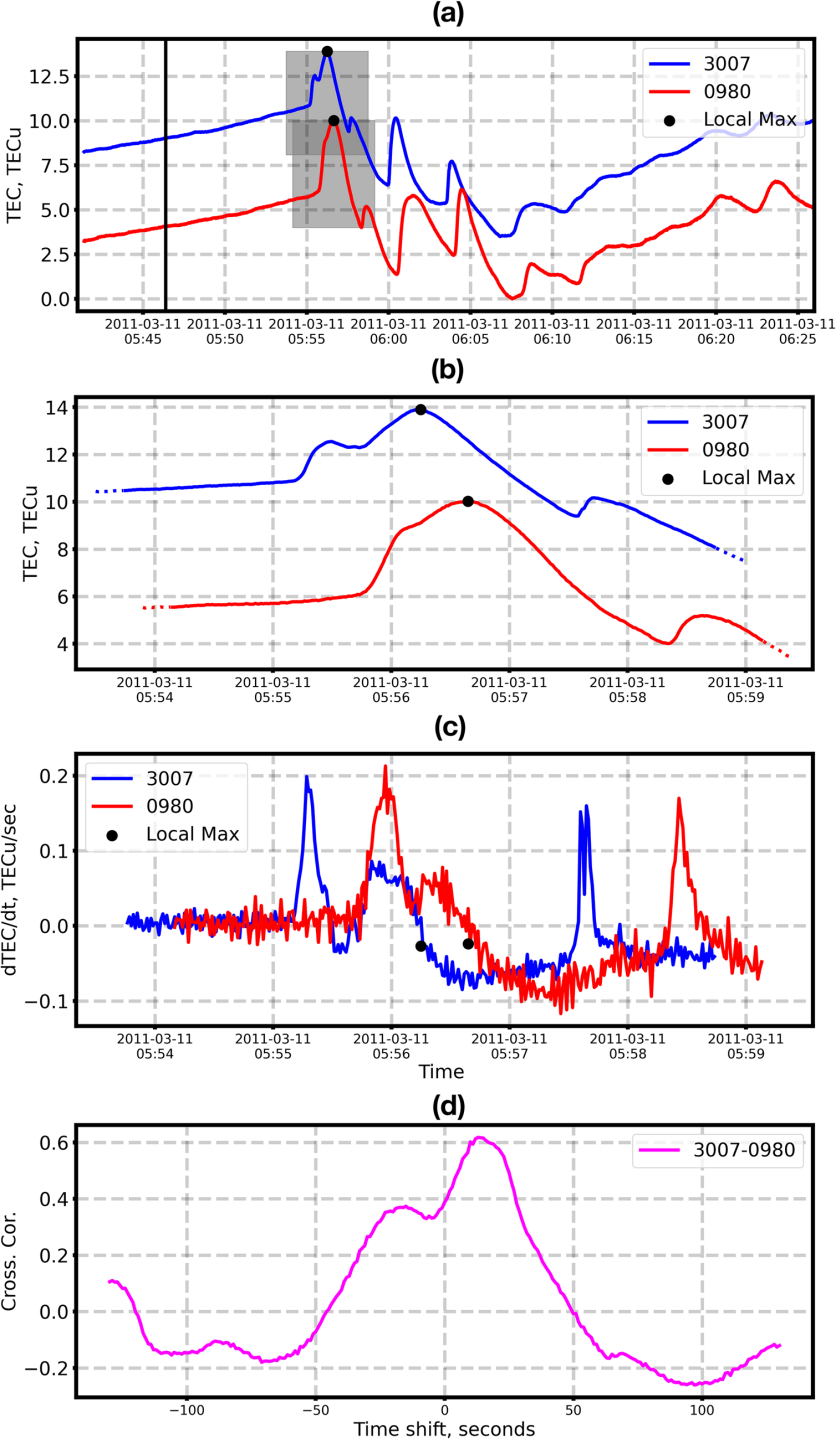
(**a**) Variations of slant TEC registered by GPS satellite 26 at stations 0980 and 3007 following the Tohoku earthquake of 11 March 2011. The earthquake time is indicated by vertical black line. Gray shaded rectangles denote 5-min time window, which is used for further cross-correlation analysis; (**b**) sTEC variations within 5-min time window; (**c**) dTEC/dt within 5-min time window. Black point shows the LMV determined from the sTEC data. The data are 1 Hz; (**d**) Cross-correlation function for the two dTEC/dt time series.

At step #2, within the window, we switch from sTEC to dTEC/dt. With such an approach, we focus on sudden strong co-seismic TEC signatures that are analogous to the peak ground displacements^36^. Figure 3a shows examples of CTID detected by GPS stations 0980, 3007 after the 2011 Tohoku-oki earthquake (1 Hz data). The co-seismic signatures in sTEC data series (panels a, b) are quite significant, however, the presence of the trend makes it difficult to calculate the correlation function and the time shift between the data series that are necessary at later steps. In turn, in the dTEC/dt data series, the CTID signatures are visible, but the trend is removed (Fig. 3c). The chosen 5-min window is enough to compute the correlation, since it catches the CTID signatures and they prevail in the time span.

At step #3, we compute the cross-correlation function for two data series in order to obtain the time shifts in the signal arrivals. The latter is found based on the maximum of the cross-correlation function. In addition, the cross-correlation can correct possible errors in finding the LMV. Finally, from the obtained maximum values, it can select 3 GNSS stations for the D1- technique, as explained below in P.3.

To calculate the cross-correlation function, we use Fast Fourier Transformation (FFT), which is a rapid procedure and suitable for NRT applications. Figure 3d shows an example of the cross-correlation function between dTEC/dt data series at two GPS receivers.

The threshold for the correlated data series depends on the standard deviation of dTEC/dt series:

2 $$T = 1 - \left( {1 - K*\sigma_{1} } \right)*\left( {1 - K*\sigma_{2} } \right)$$

where $\sigma_{1} ,\sigma_{2}$—the standard deviation of dTEC/dt series at GNSS sites. The standard deviation is an indicator of data noisiness. The noisier the data the more difficult it is to detect CTID because of a lower correlation coefficient. Therefore, our approach will adaptively consider the data noise level. Another issue in determining the threshold *T* is linked with different data cadences. The dTEC/dt values will increase with data cadence. Consequently, to adapt the threshold estimation to different data sampling, we introduce a normalizing coefficient *K*. For 1-Hz data, the *K* is chosen to be 10 TECu^−1^ based on data analysis. Such an adaptive approach makes our method adjustable to the scale of an ionospheric response and aids to automate the triangle selection process (at a later step). It is known that smaller earthquakes generate CTID of smaller amplitudes^51^. When the response is weaker, the threshold is smaller due to the smaller standard deviation of dTEC/dt series and vice versa. (Figure S1). Setting a constant threshold may affect the results and that there is a need for an adaptive algorithm for this problem.

### Real-time estimation/determining of spatio-temporal parameters: D1-GNSS-RT method

To determine spatio-temporal parameters of CTID, such as the horizontal velocity and the azimuth of propagation, we use a so-called “D1” method. This is an interferometric approach that was introduced by Afraimovich et al*.*^37^ to analyze and detect TID, of which CTID are a subclass. Originally, this method was based on use of GPS-measurements only^37,38^. Our method works with all GNSS data, and it is real-time compatible, therefore, we refer to it as “D1-GNSS-RT”.

**Figure 4:**
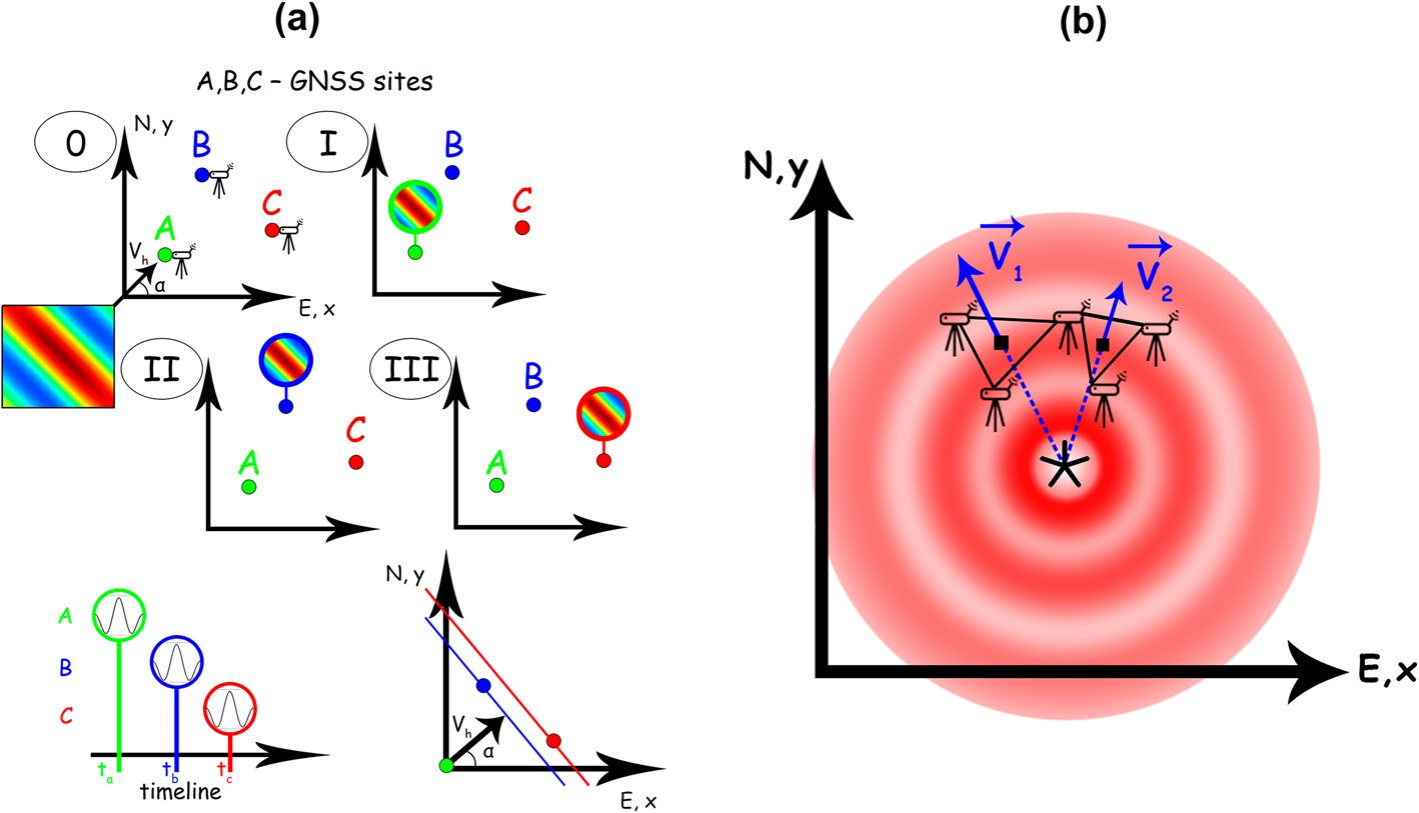
(**a**) Explanation of D1 technique**.** A, B, C—GNSS stations that are used to determine the CSID parameters: horizontal velocity (*v*_*h*_) and azimuth (*α*). 0, I, II, III mark the moments of time when the perturbation approaches the detection triangle (0) and when the perturbation is detected at points A, B, and C, respectively. The wavefront is considered to be plain; (**b**) Ionospheric localization of CTIDs based on the known location and values of two velocity vectors V_1_ and V_2_ as determined by using the D1-method

The disturbances detected by a system of three spatially separated receivers, that act as an interferometric system, are considered to be parts of the same wavefront (Fig. 4a). Then, by analysing the wave characteristics (such as phase, frequency, signal amplitude) of the observed disturbances, we determine the time shift between CTID arrivals at the detection “triangle”. Three assumptions are used in the subsequent calculations: (1) the wave front is plane, i.e., the distance between the receivers is less than the horizontal dimensions of CTID; (2) the wave front is homogenous; (3) the CTID propagate horizontally i.e. the GNSS-receivers detect the perturbations at the same altitude (*Hion*).

At the “0” time moment, a disturbance with horizontal velocity *v*_*h*_ and azimuth *α* is approaching the “A–B–C” interferometric system. At the moment “I”, the CTID is detected by the receiver “A”, and it is further moving to other receivers of the system. It is important to note that the consideration of the wave front as plane and homogeneous means that both *v*_*h*_ and *α* would not change when the CTID arrives at the other points of the given system. Garrison et al*.*^39^ showed the correctness of such an assumption for small-scale (3–10 min) TIDs, based on the dense network of receivers in the limited space. At moment “II”, the CTID has already passed receiver “A”, and arrived at receiver “B”. At “III”, the CTID arrives at receiver “C”. Only after this step, one can compute the characteristics of the perturbation. The velocity *v*_*h*_ and the azimuth *α* are then estimated by using the following formulas^40^:

3 $$u_{x} = \frac{{x_{A} *y_{C} - x_{C} *y_{A} }}{{y_{C} *\left( {t_{A} - t_{B} } \right) - y_{A} *\left( {t_{C} - t_{B} } \right)}}$$

4 $$u_{y} = \frac{{x_{A} *y_{C} - x_{C} *y_{A} }}{{x_{A} *\left( {t_{C} - t_{B} } \right) - x_{C} *\left( {t_{A} - t_{B} } \right)}}$$

5 $$v_{h} = \frac{{u_{x} *u_{y} }}{{\sqrt {u_{x}^{2} + u_{y}^{2} } }}$$

6 $$\tan \alpha = \frac{{u_{y} }}{{u_{x} }}$$

For better spatial representation, the location of the obtained horizontal velocity vector is placed at the point with the first arrival of the disturbance (point A in Fig. 4a). While, in the temporal domain, the obtained velocity is linked with the arrival time of the disturbance at point C.

As mentioned before, the D1-method is only applicable to a TID with a plain waveform. It is known however that, in most cases, the wave front of CTID is circular (e.g.,^5,41^). Therefore, the farther are the stations from one other, the worse is the plain wave condition fulfilled. Also, larger distance between the stations will lower the maximum of the cross-correlation function. Consequently, the D1-GNSS-RT can only be used on a very small segment of the circular wavefront. This limitation requires additional analysis of the positions of the A, B, C receivers with respect to the wavefront. To do that, here we use the cross-correlation function that is the criterion of the similarity of multiple data series. It should be noted that the waveform of the CTID largely depends on the conditions of observations, such as magnetic field configuration in the epicentral area, geometry of GNSS-sounding and the background ionization (e.g.,^41–44^). Therefore, only perturbations registered close to one another will have similar waveforms.

### Localization of the source of ionospheric disturbances

The velocity field obtained by the D1-GNSS-RT method can further be used to locate the source of CTID. The source is defined as a point in the ionosphere where the CTID is generated and starts to propagate horizontally outward from the source. We switch to Latitude–Longitude coordinate system, where x-axis is directed from West to East and y-axis is directed from North to South (Fig. 4b). We take the azimuths (α_i_) and the values (v_i_) of the velocities, as well as the coordinates ($lon_{0i}$ and $lat_{0i}$) of the velocity “vectors” from the output of the D1-GNSS-RT. This gives us a linear system, where the coordinates ($lon_{0}$ and $lat_{0}$) of the source of ionospheric disturbances are unknown. There are two additional restrictions on the system solutions: (1) the horizontal distance between the vectors should be less than 50 km and (2) the difference in the arrival times between points A–B and A–C should be less than 30%. These restrictions are thought to avoid the location of velocity vectors to be on the same segment of the CTID wavefront in order to fulfill the condition of the plain wavefront.

For one velocity vector the distance to the source is defined by the following equation (Fig. 4b):

7 $$lon_{0} - lon_{0i} = \tan \left( {\alpha_{i} } \right)*(lat_{0} - lat_{0i} )$$

where $lon_{0}$ and $lat_{0}$—the coordinates of the source, $lon_{0i}$ and $lat_{0i}$—that of the given velocity vector, *α*_*i*_—the azimuth of the velocity vector. Similarly, for two vectors we obtain:

8 $$\left\{ {\begin{array}{*{20}l} {lon_{0} = \tan \left( {\alpha_{1} } \right)*(lat_{0} - lat_{01} ) + lon_{01} } \hfill \\ {lon_{0} = \tan \left( {\alpha_{2} } \right)*(lat_{0} - lat_{02} ) + lon_{02} } \hfill \\ \end{array} } \right.$$

Based on the system above, the coordinates of the intersection of the two vectors can be estimated as:

9 $$\left\{ {\begin{array}{*{20}l} {lat_{0} = \frac{{\left( {lon_{02} - lon_{01} } \right) + \left( {lat_{01} *\tan \left( {\alpha_{1} } \right) - lat_{02} *\tan \left( {\alpha_{2} } \right)} \right)}}{{\tan \left( {\alpha_{1} } \right) - \tan \left( {\alpha_{2} } \right)}}} \hfill \\ {lon_{0} = lon_{01} + \tan \left( {\alpha_{1} } \right)*(lat_{0} - lat_{01} )\; \;or\;\;lon_{02} + \tan \left( {\alpha_{2} } \right)*(lat_{0} - lat_{02} )} \hfill \\ \end{array} } \right.$$

Once the source location is known, along with the velocity vector location and its value, the onset time of the source is estimated as follows:

10 $$t = t_{i} + \Delta t_{i}$$

where $t_{i}$ is the time of the velocity vector and $\Delta t_{i}$ is defined by:

11 $$\Delta t_{i} = \frac{{Dist\left( {lon_{0} ,lat_{0} ,lon_{0i} ,lat_{0i} } \right) }}{{v_{i} }}$$

where $Dist\left( {lon_{0} , lat_{0} ,lon_{0i} , lat_{0i} } \right)$ is the distance between the source location and the velocity vector location. If the difference in determination of the source onset time from the two given velocities is less than the sampling interval, we consider this pair of velocities as a possible solution for a specific moment of time and location of the source.

## Results

**Figure 5:**
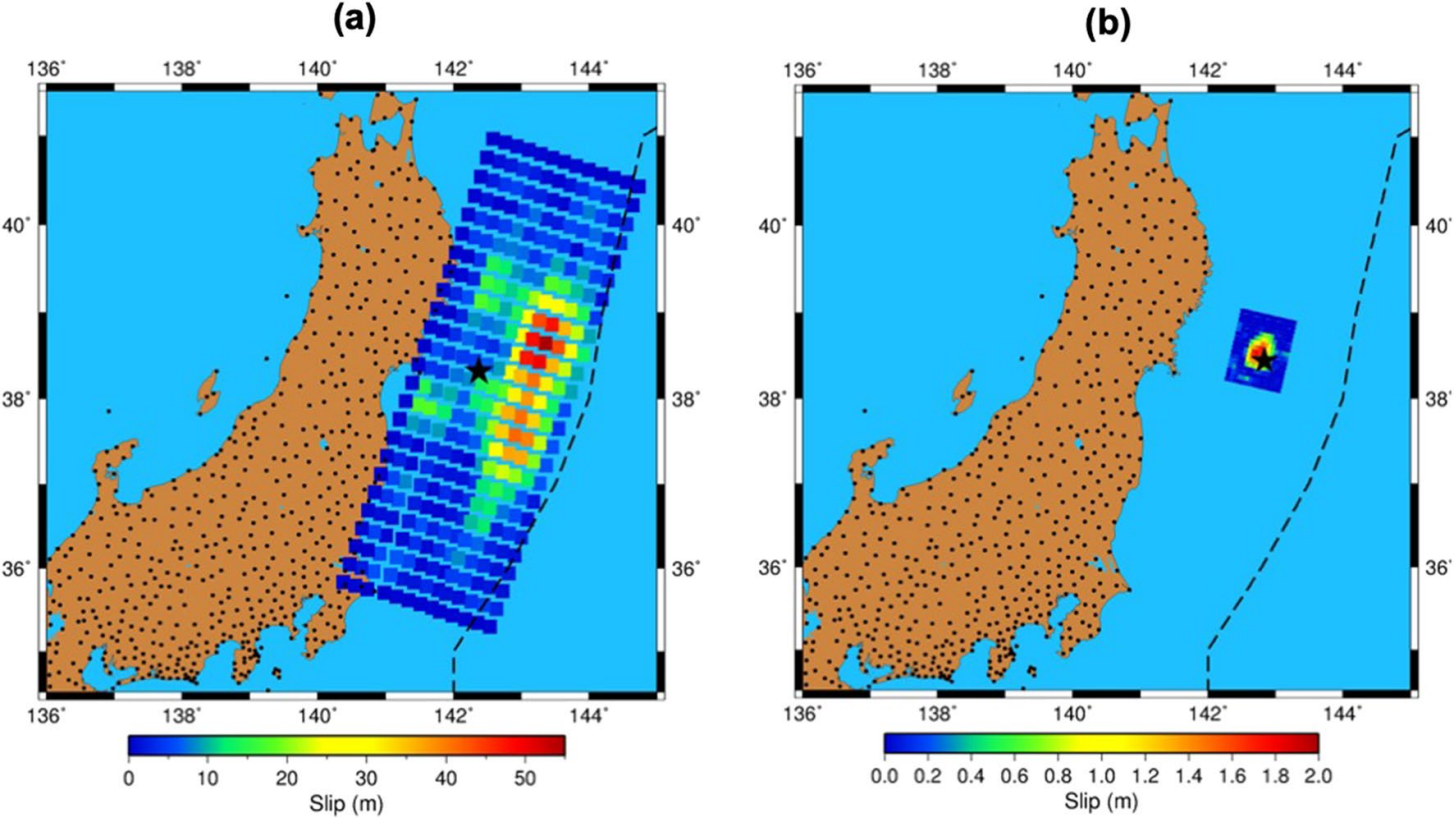
Maps for the Mw9.0 Tohoku earthquake of 11 March 2011 (**a**) and the M7.3 Sanriku earthquake of 9 March 2011 (**b**). Black star shows the epicenter, black dots show GPS receivers, and the colored squares depict the amplitude of the co-seismic slip that occurred due to the earthquakes as calculated by the NEIC USGS^41^. The corresponding color scale is shown on the bottom. The dotted curve shows the position of the Japan Trench. The maps were plotted by using GMT6 software^48^.

We apply our newly developed methods to the cases of two shallow (~ 32 km) earthquakes that occurred in March 2011 off the east coast of Honshu, Japan. The first one is the great M9.1 Tohoku-oki earthquake. According to the US Geological Survey (The National Earthquake Information Center (NEIC); http://earthquake.usgs.gov), the epicenter of this earthquake was located at 38.322° N and 142.369° E (Fig. 5a), and the onset time was estimated at 05:46:26 UT. The rupture lasted about 180 s, and caused significant co-seismic cumulative slip with the maximum of 56 m on the north-east from the epicentre (Fig. 5a)^45^. Several research groups pointed out that the Tohoku earthquake slip consisted of 2 or 3 “segments” (e.g.,^46,47^), that present multiple sources for the ionospheric disturbances (e.g.,^19^).

The second event is the M7.3 Sanriku-oki earthquake that occurred 55 h before the Tohoku earthquake (i.e., on 9 March) and is often referred to as the Tohoku foreshock. According to the USGS, the rupture started at 02:45:20 UT at the epicentre with coordinates: 38.435° N, 142.842° E (Fig. 5b). This smaller event lasted 30–40 s and provoked a 2 m co-seismic slip on the north-west from the epicentre (Fig. 5b)^49^.

To analyze the CTID activity, in both cases, we apply our method to 1 Hz GNSS ionospheric data from the Japan GNSS Earth Observation Network (GEONET, https://www.gsi.go.jp).

### The velocity field and ionospheric localisation of the 2011 M9.1 Tohoku-oki earthquake

The ionospheric response to the Tohoku earthquake was studied in detail by numerous research teams (e.g.,^5,6,14,15,19,50^). As shown in Fig. 3, the near-field TEC response showed very complex waveforms, with several peaks in TEC data. The amplitude of this response was also quite significant as compared to other earthquakes and was detected by ten GPS satellites^5,6,34,35^. Here we work with data of GPS satellite 26 that showed the largest and the clearest co-seismic signatures.

**Figure 6:**
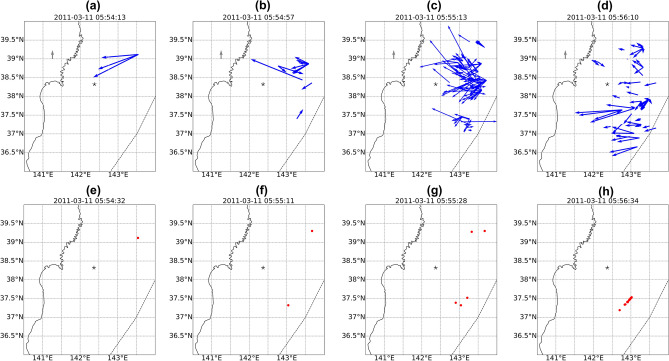
(**a-d)** CTID velocity field calculated from the first CTID detected by GPS satellite PRN 26 after the Tohoku earthquake. The dotted curve shows the position of the Japan Trench, black star depicts the epicenter. The gray arrow corresponds to 1.1 km/s; (e–h) localization of the seismic source as estimated from the first velocity vectors shown on panels (a–d).

The CTID velocity field maps for the first CTID arrivals following the Tohoku earthquake are shown in Fig. 6a–d, and the localization results are shown in Fig. 6e–h. It should be noted that, in principle, we can calculate the CTID characteristics for multiple periods of time, as long as the perturbations are detected. For the Tohoku event, instantaneous velocity maps for the first 2 min of CTID detection can be found in Animation S1 (available as supplementary material), and the localization results are shown in Animation S2 (supplementary material). Figure 6a shows the first velocity vectors at 05:54:13UT, i.e. 487 s after the earthquake onset time, on the north-east from the epicenter. The first vectors are directed south-westward, and the first points have the velocities of about 4 km/s. Such velocity values might correspond to the propagation of the primary (P-) seismic waves (i.e., the rupture propagation), or to the propagation of the Rayleigh surface waves. These first velocity vectors give the first source location at the point with coordinates (38.18; 143.55) (Fig. 6e). At 05:54:57UT, one can see further development of the CTID evolution within the source area, with smaller velocities. In addition, we notice the occurrence of the second source on the south-east from the epicentre (Fig. 6b, f). Further, one can clearly see the occurrence of the second segment of the source on the south-east from the epicenter (Fig. 6d, g). At 05:56:10UT, we observe further evolution of CTID, and westward propagation of CTID with velocities from 600 m/s to ~ 3 km/s. This range of velocities was previously observed for the CTID generated by the Tohoku earthquake (e.g.,^5,6,14^).

The CTID propagation speed can be verified by plotting so-called travel-time diagrams (TTD), that present 3-D diagrams with the distance from the source versus time after the source onset, and the amplitude of CTID is shown in color. TTD also enable to confirm the correlation of the observed perturbations with the source. In retrospective studies, a band-pass filter was applied in order to better extract the co-seismic signatures and to clearly see the correlation with the source. In NRT mode, and with the impossibility to use such a filter, we suggest using dTEC/dt parameter, and we call such diagrams near-real-time TTD (NRT-TTD). This is the first NRT-compatible method proposed for obtaining the TTD. As a source, at the first approximation, we can take the epicentre position that should be known from seismological data several minutes after the earthquake. However, the epicentre is the point where the rupture starts, and its position does not always correspond (especially for large earthquakes) to the position of the co-seismic crustal uplift that generates CTID as well as tsunamis. The problem lies, however, in the fact that in NRT, it is very difficult to know the position of the uplift or the slip. Therefore, we can take the position of the source estimated from our ionospheric methods.

**Figure 7:**
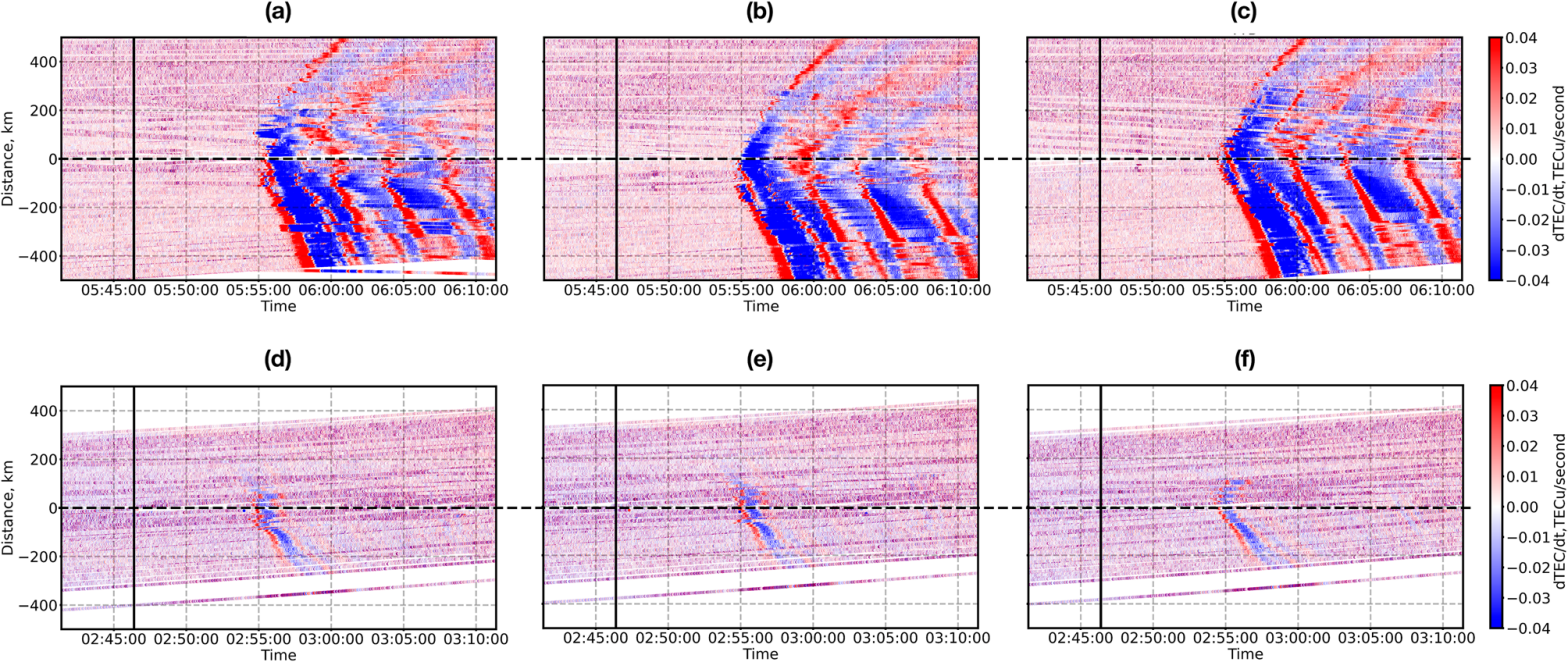
Near-real-time travel time diagram (NRT-TTD) plotted by using dTEC/dt data for the Tohoku (**a**–**c**) earthquake (satellite G26) and Sanriku (**d**–**f**) earthquake (satellite G07). In panels (**a**, **d**) the distance is calculated with respect to the earthquakes’ epicenters as estimated by the USGS, in panels (**b**, **e**)—with respect to the maximum co-seismic uplifts; (**c**, **f**)—with respect to the ionospheric localization as shown in Fig. 5d, e and 6d, e. The color scale is shown on the right.

The NRT-TTD for the Tohoku event, G26 satellite, plotted for the source located at the epicentre, the center of the maximum slip (38.64; 143.35) and the “ionospheric source” (37.944; 143.153) are presented in Fig. 7a–c, respectively. It should be noted that the Tohoku earthquake produced significant displacement of the ground on a large area (the approximative fault size is about 300*80 km) and, strictly speaking, taking a single point as the source is an approximation. However, we proceed with such an assumption to plot the NRT-TTD. The correlation is seen when CTID propagates “linearly” from the source. Comparison of Fig. 7a–c reveals that the best correlation is obtained for the slip maximum (Fig. 7b) and for the ionospherically-determined source (Fig. 7c). While, the perturbation is not well-aligned when the diagram is plotted with respect to the epicentre (Fig. 7a). The propagation speed of the observed CTID can be estimated from the slopes on the TTD. We find the speeds to be ~ 2.3–2.6 km/s, which is in line with previous retrospective observations for the ionospheric response to the Tohoku earthquake (e.g.,^5,6,14,15^).

### The velocity field and ionospheric localisation of the 2011 M7.3 Sanriku-oki earthquake

Ionospheric response to the Sanriku earthquake was studied previously by Thomas et al*.*^53^ and Astafyeva and Shults^32^. The co-seismic TEC signatures were detected by satellites G07 and G10. Here we only focus on CTID registered by GPS satellite G07. Contrary to the CTID generated by the Mw9.0 Tohoku earthquake, the ionospheric TEC response to this smaller earthquake presented the commonly known N-wave signatures with smaller amplitudes. However, even despite the smaller amplitude of CTID, our method detects these disturbances.

**Figure 8:**
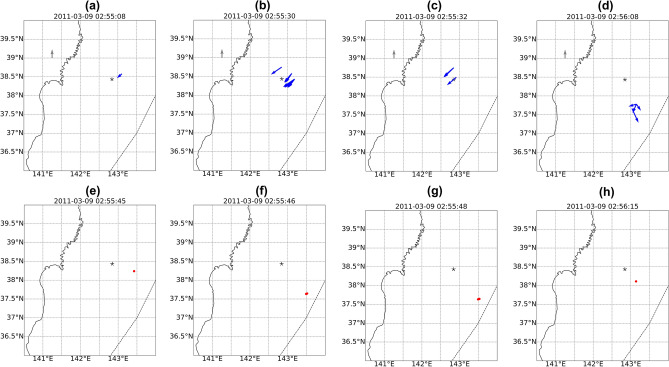
(**a**–**d**) CTID velocity field calculated from the first CTID detected by GPS satellite PRN 07 following the Sanriku earthquake. The dotted curve shows the position of the Japan Trench, black star depicts the epicenter. The gray arrow corresponds to 1.1 km/s; (**e**–**h**) localization of the seismic source as estimated from the first velocity vectors shown on panels (**a**–**d**).

The instantaneous velocity field maps are presented in Fig. 8a–d. One can notice that the picture of the velocity field for the CTID generated by the Sanriku event is much simpler that the one for the Tohoku event. The first velocity vector is shown at 02:55:08UT, i.e. 588 s after the earthquake onset time. At that instant, the CTID starts to propagate south-westward at the velocity of about 850 m/s (Fig. 8a). Within the next minute, we observe south-westward propagation of ionospheric disturbances at ~ 850–1100 m/s (Fig. 8b, c). At 02:56:08UT, we observe further southwestward propagation of CTID (Fig. 8d). From these first velocity fields, we estimate the location of the source to be on the south-east from the epicentre (Fig. 8e–h). Overall, one can notice significant difference in the velocity field and CTID evolution during this smaller earthquake. The CTID have lower velocities, and the velocity field is much less complex as compared to the Tohoku earthquake.

The corresponding RT-TTD calculated with respect to the epicentre, the maximum slip point (38.5; 142.7), and the ionospherically determined (38.335; 143.442) source are presented in Fig. 7d–f, respectively. The best alignment is achieved for the ionospheric source (Fig. 7e), where we also see concurrent northward and southward propagation from the source. While, for the two other sources one cannot clearly see this effect (Fig. 7a, e). Therefore, our results suggest that the source was located on the south-east from the epicentre. The worst alignment is obtained for the epicentre as the source of CTID (Fig. 7d). The CTID propagation speed is estimated to be 1.2–1.6 km/s, which is close to the estimation in after-earthquake analysis by Astafyeva and Shults^32^.

## Discussions

Above we demonstrated the possibility to calculate in NRT spatio-temporal characteristics of CTID on the example of two earthquake events that occurred in Japan in March 2011. For both earthquakes, we also localized in NRT the source of the observed CTIDs. It should be reminded that the CTID coordinates and, consequently, the estimated position of ionospheric sources will change if we vary the altitude of detection *Hion*. In this work, we took *Hion* = 250 km, which is close to the ionization maximum in the epicentral areas during the earthquakes, and is the right choice from a physical point of view. However, recently it has been suggested that the actual GNSS detection of CTID may take place at lower altitudes^19,32,53^. Therefore, strictly speaking, the *Hion* should be determined each time for the correct estimation of the CTID coordinates. Our method is fully operational independently on the *Hion* value, however, its results and the accuracy of the ionospheric source localization might be improved if/when we know the real *Hion*. Determining the exact altitude of detection is out of the scope of the current work.

Here we used 1 Hz GNSS TEC data from the Japanese network of GPS receivers GEONET, i.e. a network with good spatial coverage with 20-km distance between the receivers, and we demonstrated that in such observational conditions, our NRT-compatible methods provide good results both in terms of the source localisation and determining of CTID spatio-temporal characteristics. In our method, the accuracy of localisation seems lower than that by seismic stations that invert the position of the epicentre based on detection of seismic waves. The seismic source can also be localized by other non-seismic instrumentation, such as by balloon pressure sensors via detection of infrasound signals due to earthquakes. For instance, Krishnamoorthy et al*.*^54^ showed that the source can be localized with 90% probability within an ellipse with a semimajor axis approximately 80 m under the perfect conditions. They used 26 shots that is equal to the usage of a 26-balloon array to solve this task. It should be noted, however, that this result was obtained by a-posteriori analysis, therefore it might be quite challenging to repeat such quality in NRT.

Further, we discuss how lower or much lower spatial and temporal resolutions of GNSS ionospheric data could affect the output of our methods. Also, the accuracy of estimation of the velocities and the source location should be determined.

**Figure 9:**
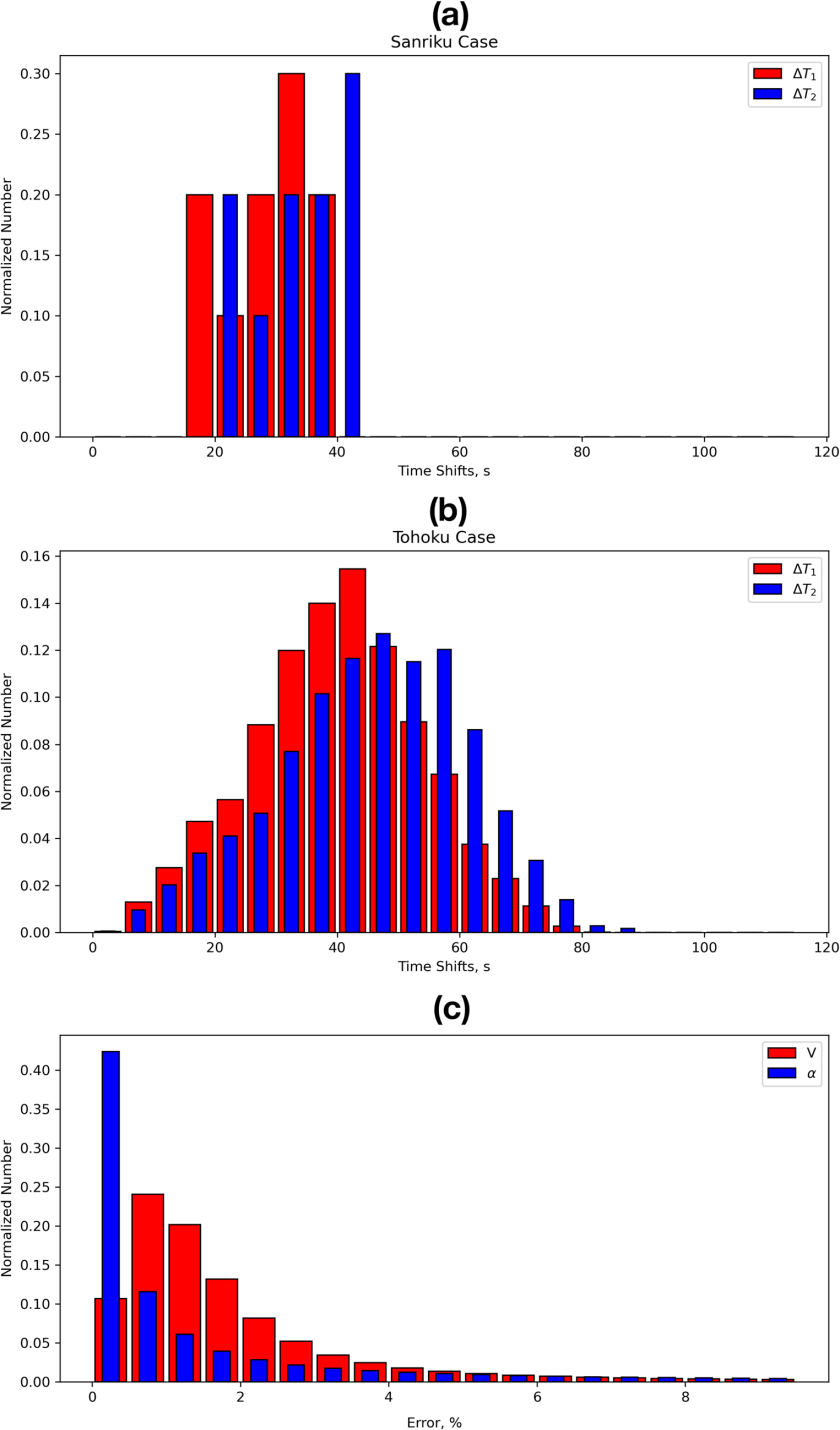
(**a**–**b**) Distribution of normalized number of the time shifts between points A–B (red, ΔT_1_) and A–C (blue, ΔT_2_) of a triangle for the Tohoku (**a**) and Sanriku (**b**) earthquakes; (**c**) impact of an error of ± 0.5 s on arrival times affects the computation of the velocities values and azimuths.

With regards to the data sampling, for both earthquakes, we tested our methods on 30-s data that are available from the GSI (http://datahouse1.gsi.go.jp/terras/terras_english.html). We have found that such a resolution is not enough because of two main reasons. First, fewer data within the selected window duration of 5 min will smooth the dTEC/dt values, which, in turn, will erase the specific features of CTID that characterize different segments of the wavefront. As mentioned before, the D1-GNSS-RT method can only be used on a small part of a wavefront, because it is only applicable to the plain wave. Therefore, with 30-s data sampling, it is difficult to control this condition in terms of the correlation between data series, especially for smaller earthquakes, for which the response is smaller in amplitude and duration^51^. Second, 30-s data rate will introduce ± 15-s error in the LMV determining within the window, and, consequently, it will lead to errors in the arrival time at each point of a triangle. The impact of such ± 15-s error can be seen in Fig. 9a, b, where we present the normalized number of the time shifts between points A–B (red, ΔT_1_) and A–C (blue, ΔT_2_) of a triangle for the Tohoku (a) and Sanriku (b) earthquakes. For both events, the distribution of ΔT_1_ and ΔT_2_ have the same shape and look quite similar, but are shifted for ~ 5 s. This emphasizes the fact that a CTID arrives at points B and C at close moments of time, that is only possible if the arrivals belong to the same segment on a circular disturbance wavefront. One can also notice that, for both events, the majority of arrivals are registered within a narrow period of time, 20–40 s for the Sanriku event (Fig. 9a) and 25–60 s for the Tohoku event (Fig. 9b). This means that lower time steps in data will lead to errors in the correct detection of the moment of arrival would occur, and, consequently will eventually impact the velocity values and the azimuths.

**Figure 10:**
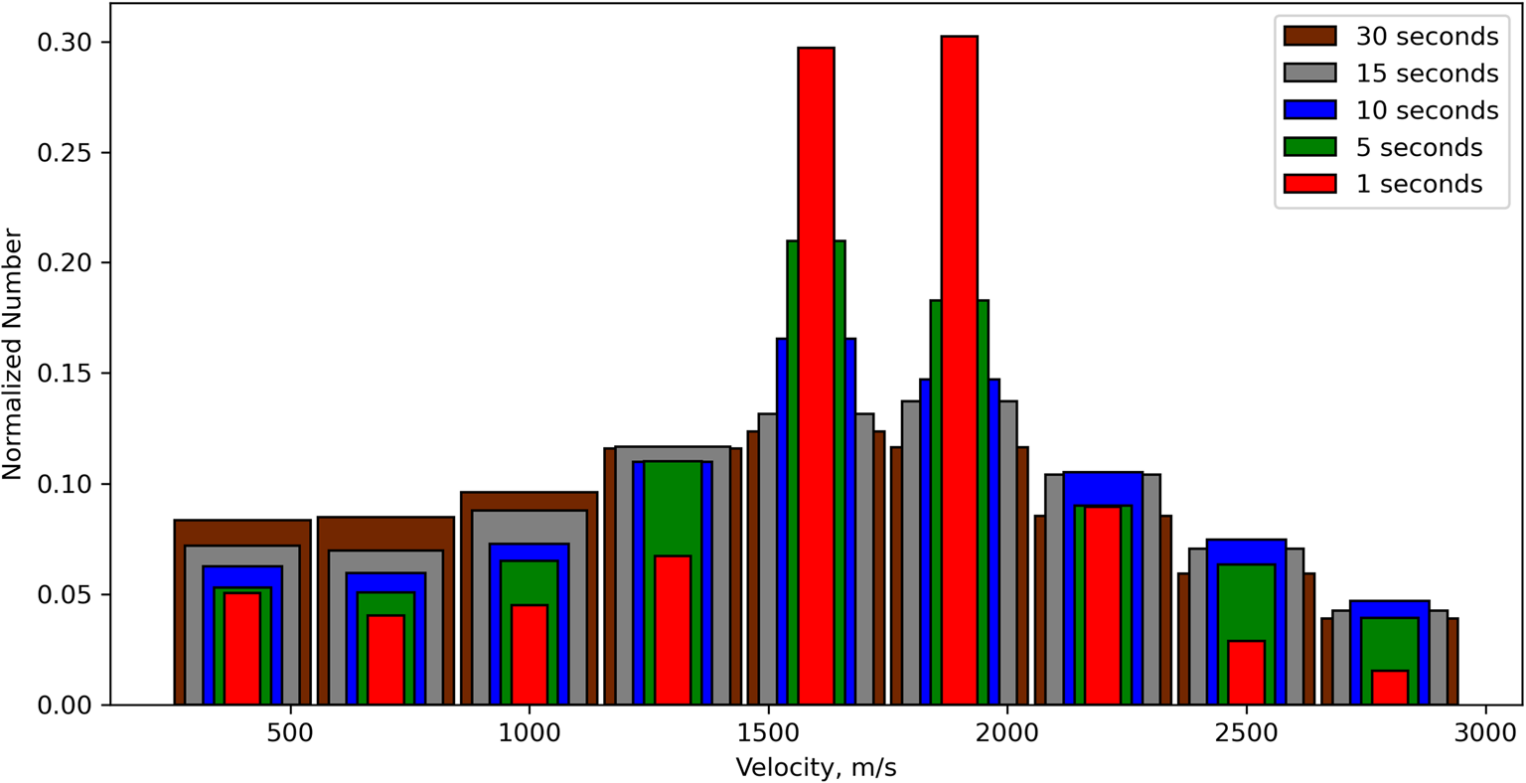
Distribution of velocity values calculated from data of different temporal cadences: 1-(red), 5-(green), 10-(blue), 15-(gray), 30-(brown) seconds.

To further analyze the applicability of our method to lower cadence data, we downsampled the initial 1 Hz data to 5-, 10- and 15-s cadence. Figure 10 shows how different data cadences impact the distribution of calculated velocities. One can see a significant difference in the results for 1 s and 30 s data. Therefore, for better performance of our methods we suggest the use of GNSS-data with 1 Hz sampling.

With respect to the accuracy of our method, we analyzed how an error of ± 0.5 s in arrival times affects the computation of the velocity values and azimuths. The normalized number of error cases versus the absolute error percentage is shown in Fig. 9c. One can see that ~ 80% of both velocities and azimuths have less than 2.5% of errors and ~ 95%—less than 5%. These results also confirm the advantage of high-rate data.

**Figure 11:**
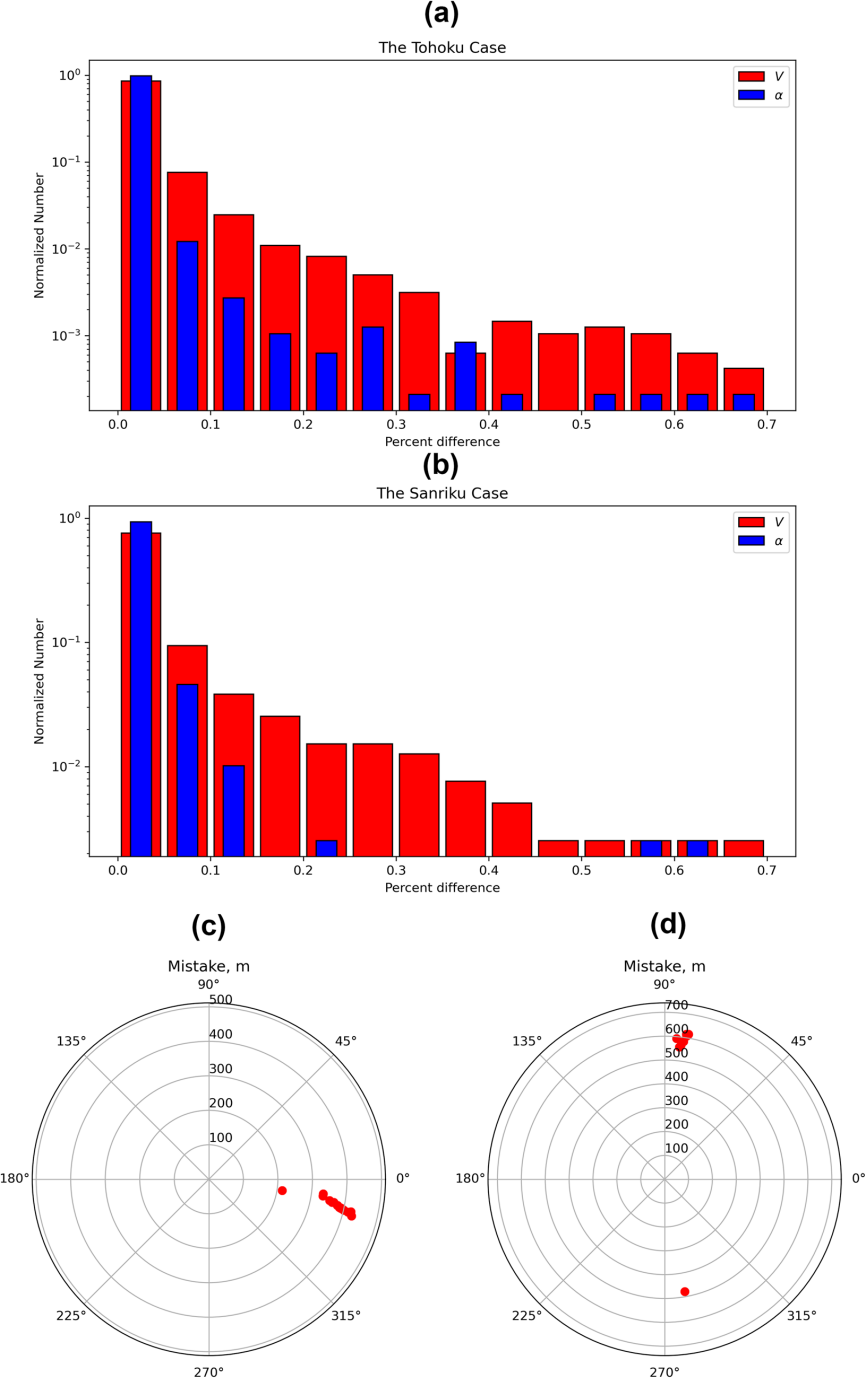
Accuracy comparison based on different sources of the orbits: navigational RINEX file and ultra-rapid orbits. Panel (**a**)—distribution of percentage difference of amplitude and azimuth of propagation for the Tohoku case (y-axis logarithmic scale); panel (**b**)—distribution of percentage difference of amplitude and azimuth of propagation for the Sanriku case (y-axis logarithmic scale); panel (**c**)—radar diagram of source location difference for the Tohoku case; panel (**d**)—radar diagram of source location difference for the Sanriku case.

The use of different orbital information can impact the accuracy of our method, because the coordinates of CTID depend on the position of a satellite as well as of that of a GNSS station. The commonly used ephemerides are those transferred in the RINEX navigation file. Alternatively, ultra-rapid orbits can be used. We compared the amplitude and direction of the obtained velocity vectors based on ultra-rapid orbits with those calculated based on the use of the RINEX navigation files (Fig. 11a, b). Then, we computed source locations based on these velocities and estimated the error in position (Fig. 11c, d). This analysis was made both for the Tohoku and the Sanriku cases. One can see that the majority of both velocities and azimuths have less than 0.05% of differences. This fact can be explained by the high quality (cm-accuracy) of the real-time IGS products^55^. However, the radar diagrams of error positioning show worse results (Fig. 11c, d).

**Figure 12:**
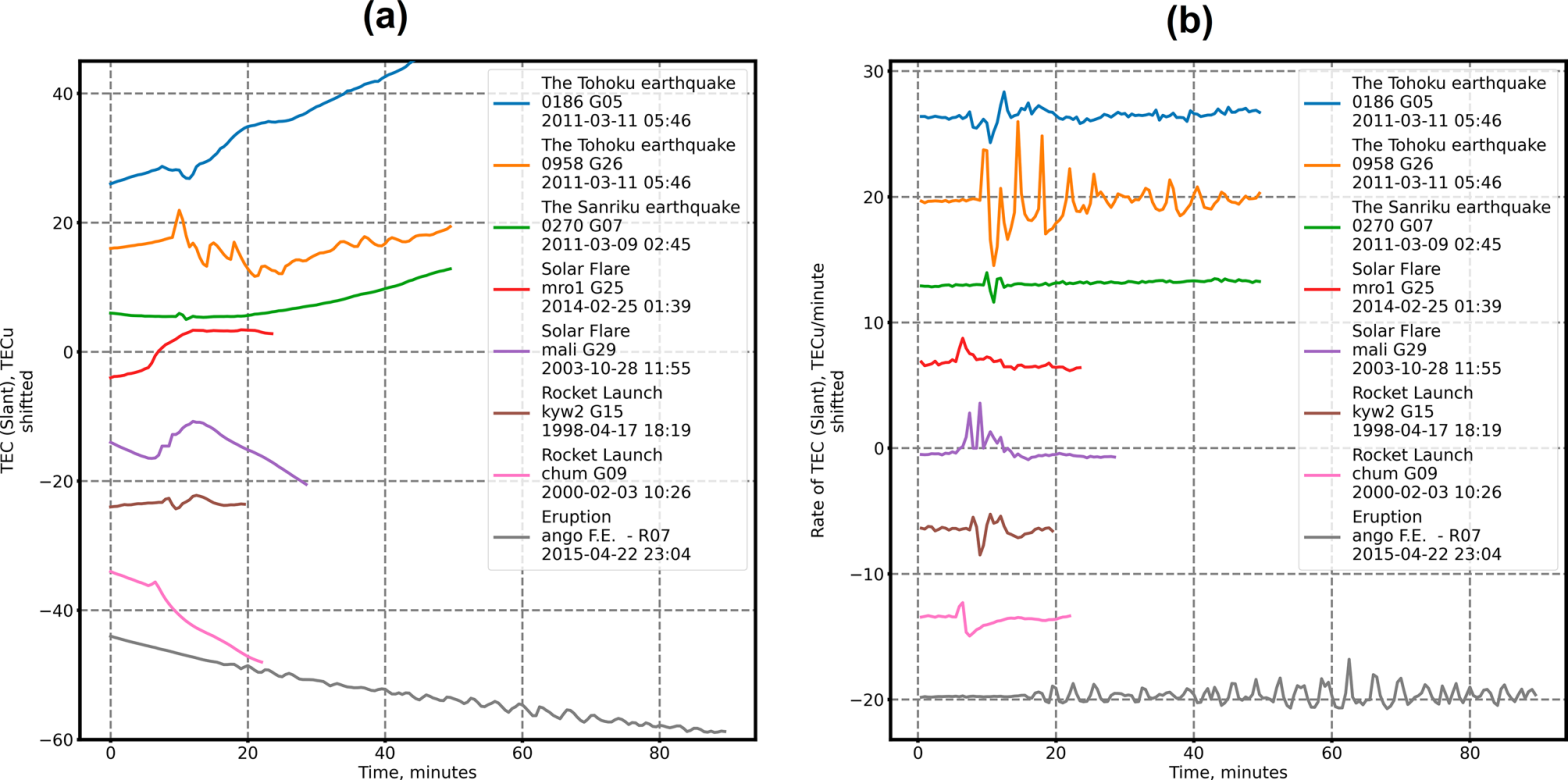
Examples of TEC disturbances of different origin that are detectable by our approach. Panel (**a**)—slant TEC values characterized by high changes, panel (**b**)**—**rate of TEC of the exact data series.

Finally, we would like to note that our methods can be used for detection of TID of other origins in addition to CTID and, therefore, it is useful for real-time Space Weather applications. The D1-GNSS-RT will automatically catch all CTID and TID with high dTEC/dt values, where the maximum disturbance amplitude exceeds the noise level by at least 4 times (Figure S4a). Such disturbances could be generated by acoustic or gravito-acoustic waves (earthquakes, volcanic eruptions, rocket launches), or by enhanced EUV radiation (solar flares) that produces rapid growth of the ionization in the ionosphere (Fig. 12). It should be emphasized that for the detection, the absolute amplitude of CTID and TID is less important than the dTEC/dt. For instance, it is known that smaller earthquakes generate smaller disturbances in the ionosphere^51,52^. Therefore, it is of interest to apply our technique to the smallest earthquake ever recorded in the ionosphere—the M6.6 16 July 2007 Chuetsu earthquake in Japan^52^. The Chuetsu earthquake produced a very small-amplitude TEC disturbance that was registered by satellite G26 and by a few GPS-stations in the near-epicentral region, and the only data available were of 30-s cadence. Unfortunately, the latter factors did not allow us to compute the velocities and the localization by using the D1-GNSS-RT technique. However, our method successfully found the LMV even for such small CTID but with sufficient dTEC/dt rate (Figure S5b, c). Also, Figure S5 demonstrates that we could track the CTID propagation with respect to the source in NRT by using our RT-TTD technique.

On the other hand, disturbances with lower sTEC derivative or/and higher noise level might appear undetectable or the D1 triangles will not be formed because of low cross-correlation between data series. For instance, we did not manage to catch CTID registered by satellites G27 (during the Tohoku earthquake) and G10 (during the Sanriku earthquake), because they had low dTEC/dt. Another example is the ionospheric response to the M7.8 2016 Kaikoura earthquake that occurred on 13 November 2016 in New Zealand, for which we also analysed high-rate 1 Hz data. The latter TEC variations presented more noise and the amplitude of the detected CTID did not grow up as fast as for the Tohoku and Sanriku cases (Figure S4). For such less pronounced disturbances, other more sophisticated methods should be developed, which is a subject of a future separate work.

## Conclusions

For the first time, we introduce a NRT-compatible method that allows very rapid determining of spatio-temporal parameters of travelling ionospheric disturbances. By using our method, one can obtain instantaneous velocity maps for ionospheric perturbations, and to estimate the position of the source. In addition, also for the first time, we present real-time travel-time diagrams. We demonstrate the performance of our methods on CTID generated by the Tohoku-oki Earthquake of 11 March 2011 and the Sanriku-oki Earthquake of 9 March 2011. We use high-rate 1 Hz GPS data from the Japan network GEONET for these two earthquakes, and we observe the evolution of the CTID over the source area as it could have been seen in real-time. We show that there is a significant difference between CTID generated by M9 and M7.3 earthquakes in terms of CTID velocities and evolution: the giant Tohoku earthquake generated a massive TEC response in both amplitude and spatial extent, and such a difference can be clearly seen in our results.

It is important to emphasize that, besides CTID, our method can detect and analyze other TID that often occur and propagate in the ionosphere. Therefore, the D1-GNSS-RT method can be used for near-real-time Space Weather applications.

## Supplementary Information

Supplementary Video 1.

Supplementary Video 2.

Supplementary Video 3.

Supplementary Video 4.

Supplementary Information 1.

## Data Availability

The data are available from the GeoSpatial Authority of Japan (GSI, terras.go.jp). http://datahouse1.gsi.go.jp/terras/terras_english.html.
