# Supplementary material for: Determining spatio-temporal characteristics of coseismic travelling ionospheric disturbances (CTID) in near real-time
Source: Sci Rep. 2021 Oct 21;11:20783. doi: 10.1038/s41598-021-99906-5 (PMC8531289; doi:10.1038/s41598-021-99906-5)
Supplement: Supplementary file 5 — Supplementary Information 1. [file 41598_2021_99906_MOESM5_ESM.pdf]

Supplementary Material

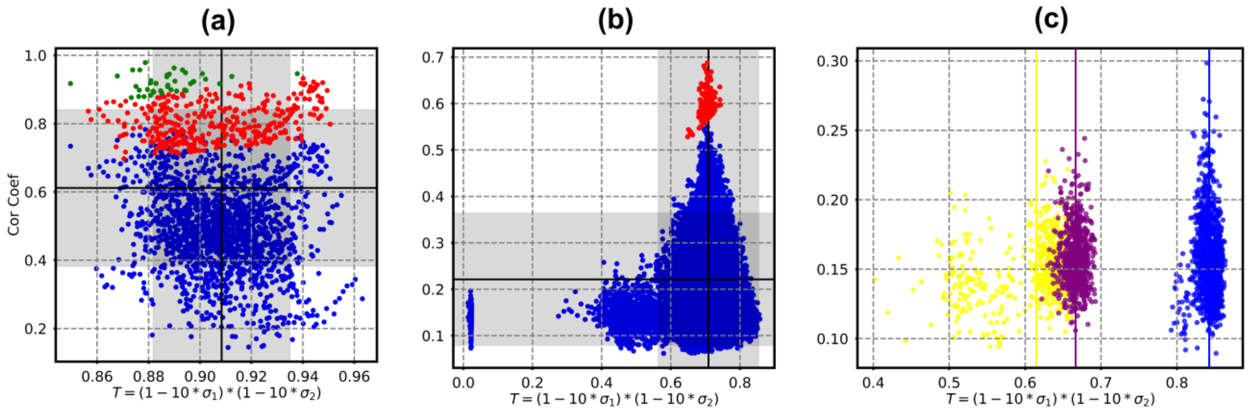

Figure S1. Distribution of relationship between the maximum value of the cross-correlation function for two dTEC/dt time series vs threshold for these data series, for the Tohoku **(a)**, Sanriku **(b)** and Kaikoura **(c)** earthquakes. On panels (a) and (b): blue dots – all values, green dots – values where the maximum value is bigger than the threshold, red dots - values where maximum value is bigger than 80% of threshold. Vertical black line corresponds to average value of threshold, horizontal black line corresponds to average value of maximum value. Gray shaded rectangles correspond to the standard deviation. On the panel (c): blue dots – all values by GPS satellite G20, yellow dots – all values by Glonass satellite R21 and purple dots – by Glonass satellite R22. In the Tohoku case (a), the ionospheric response was the best in the terms of CTIDs signatures in the output signal. This led to high values of the maximums of cross-correlation function that overpower their thresholds. The Sanriku case (b), the ionospheric response was closer to observed in many previous earthquake cases. Therefore, we shrink the threshold down to 80%.

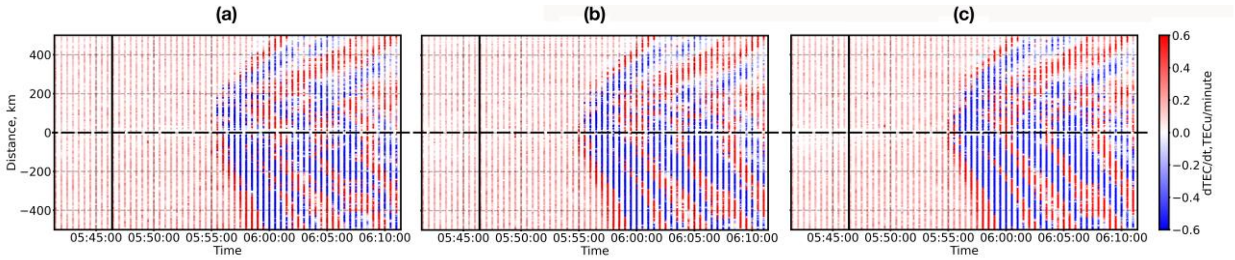

Figure S2: Examples of NRT-TTD made with 30 sec data for the Tohoku event. One can see that at thirty second resolution, the effect of the earthquake is still observable. However, due to the poorer resolution, the impact of the source position becomes less noticeable. This is another factor in favour of high-rate data.

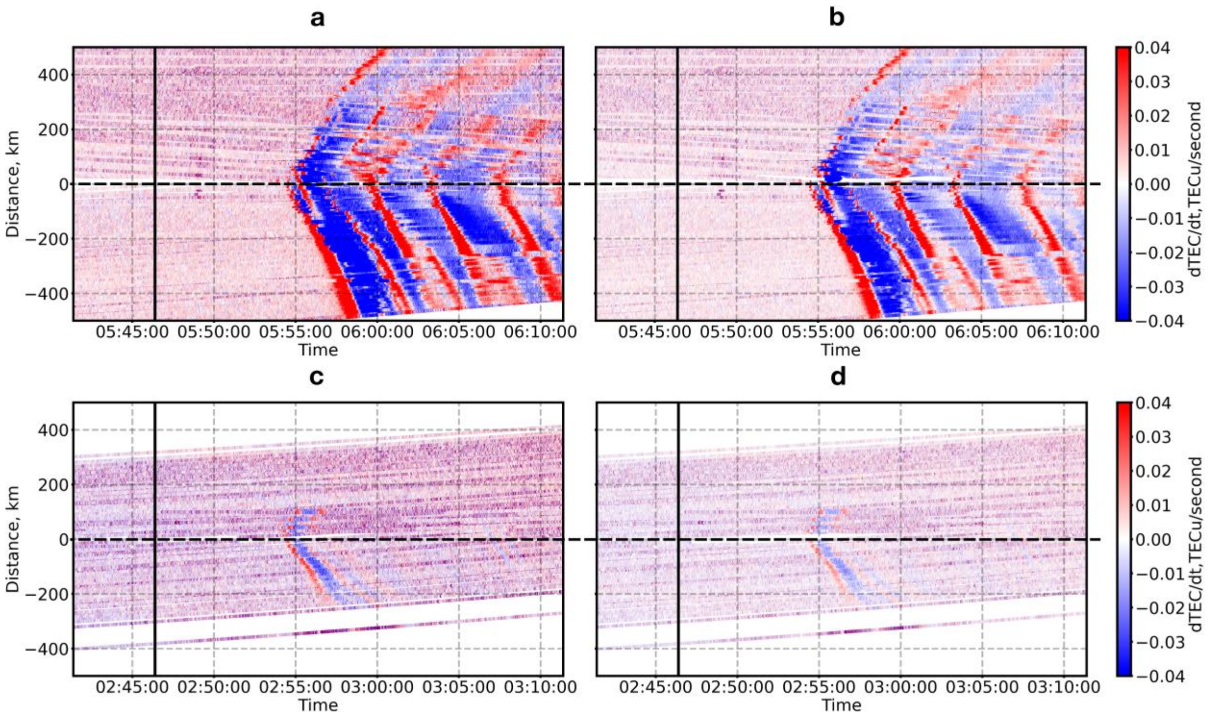

Figure S3: RT-TTD plotted for the STEC data (a, c) and vertical TEC data (b, d). The CTID signatures are quite similar, and this confirms that the observed effect is not related to distortion caused by observations at low elevation angles. The vertical TEC is calculated from the STEC by multiplying by so-called a mapping function that depends on the LOS elevation angle and the altitude of detection *Hion* [25].

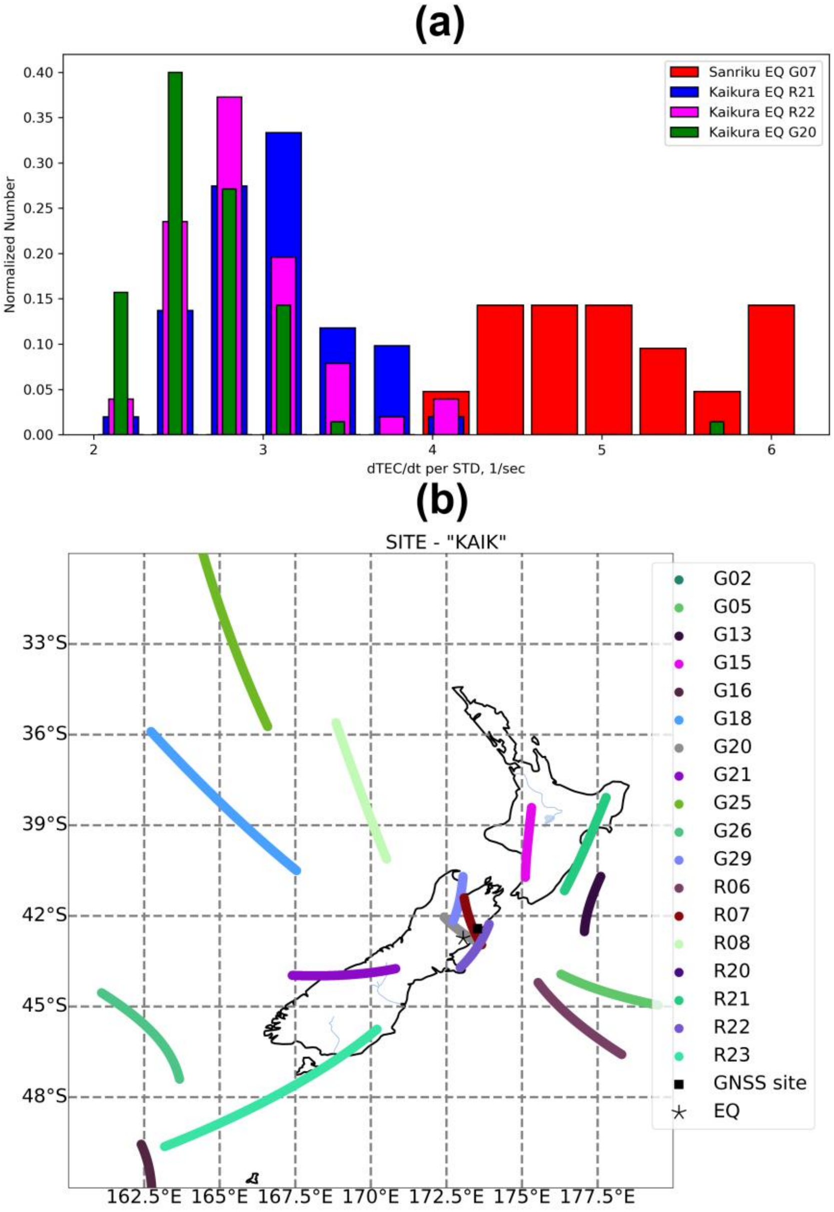

Figure S4: (a) Signal-to-noise characteristics of the ionospheric response to the M7.8 Kaikoura earthquake of 13 November 2016 as detected by GPS satellite G20 and Glonass satellites R21 and R22. The noise level is calculated as the standard deviation of dTEC/dt 5 minutes before the signal arrival. The red bars show the same characteristics for the CTID detected during the Sanriku earthquake. One can see that the Sanriku CTID had much higher dTEC/dt as compared to the Kaikoura driven CTID; **(b)** Map for the M7.8 Kaikoura earthquake of 13 November 2016. The black star shows the epicentre, the black rectangle depicts the position of GNSS-station *kaik*. Colored curves represent the trajectories of sub-ionospheric points at the altitude *Hion*=350km.

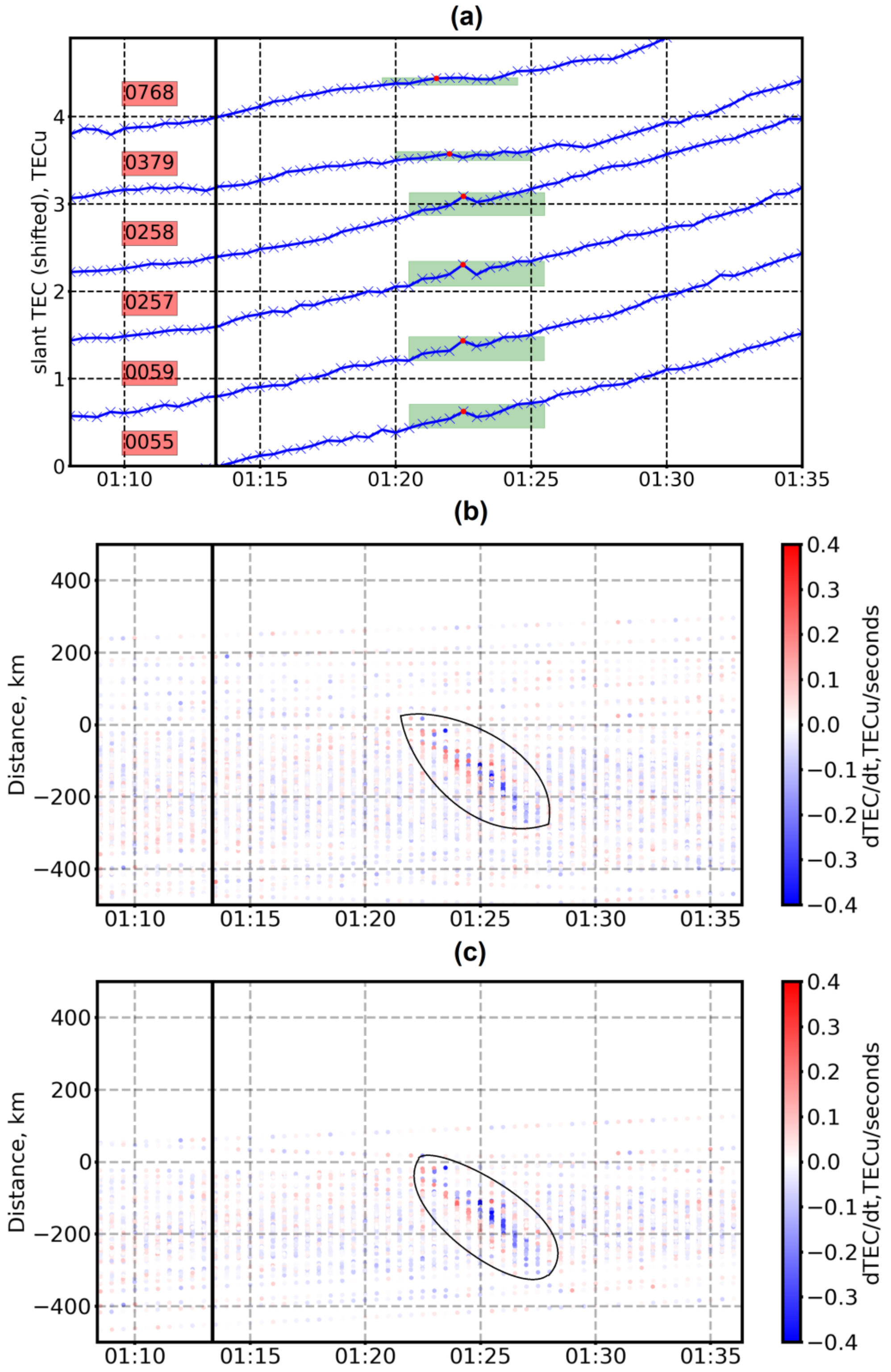

Figure S5: (a) Variations of slant TEC registered by GPS satellite 26 at GPS stations (the first arrivals in 90 seconds after the detection of the first ionospheric response) following the Chūetsu earthquake of 16 July 2007. The earthquake time is indicated by a vertical black line. Green shaded rectangles denote 5-min time window, which is used for further cross-correlation analysis; **(b, c)** NRT-TTD plotted by using dTEC/dt data. for the Chūetsu offshoreearthquake (satellite G26. In panels (b, c) The distance is calculated with respect to the earthquakes' epicenter as estimated by the USGS. All available data series were plotted for panel (b), but only data series with LMV at the output of our method were used for the plot in panel (c)

Animation S1: Velocity field for CTID generated by the M9.0 Tohoku earthquake of 11/03/2011 calculated based on high-rate 1Hz data. The dotted curve shows the position of the Japan Trench, black star depicts the epicenter. The gray arrow corresponds to 1.1 km/s.

Animation S2: Localization of the source of CTID generated by the Tohoku earthquake. The dotted curve shows the position of the Japan Trench, black star depicts the epicenter.

Animation S3: Velocity field for CTID generated by the Mw7.3 Sanriku earthquake of 09/03/2011 calculated based on high-rate 1Hz data. The dotted curve shows the position of the Japan Trench, black star depicts the epicenter. The gray arrow corresponds to 1.1 km/s.

Animation S4: Localization of the source of CTID detected following the Sanriku earthquake. The dotted curve shows the position of the Japan Trench, black star depicts the epicenter.
